# Chronotypes and disabling musculoskeletal pain: A Finnish birth cohort study

**DOI:** 10.1002/ejp.1931

**Published:** 2022-03-18

**Authors:** Eveliina Heikkala, Petteri Oura, Tuukka Korpela, Jaro Karppinen, Markus Paananen

**Affiliations:** ^1^ Center for Life Course Health Research University of Oulu Oulu Finland; ^2^ Medical Research Center Oulu University of Oulu Oulu University Hospital Oulu Finland; ^3^ Rovaniemi Health Center Rovaniemi Finland; ^4^ Rehabilitation Services of South Karelia Social and Health Care District Lappeenranta Finland; ^5^ Primary Health Care Services City of Espoo Espoo Finland

## Abstract

**Background:**

It has been suggested that chronotype, the individual preference for 24‐h circadian rhythms, influences health. Sleep problems and mental distress are amongst the greatest risk factors for musculoskeletal (MS) pain. The aims of this study were first, to explore the associations between chronotypes and MS pain, with special reference to disabling MS pain and second, to test whether mental distress and insomnia have a modifying role in the associations between chronotypes and MS pain.

**Methods:**

The dataset of 4961 individuals was composed of Northern Finns surveyed on MS pain, chronotypes and confounding factors (sex, insomnia, sleep duration, smoking, mental distress, occupational status, education level and number of coexisting diseases) at 46 years. The relationships between chronotypes (evening [E], intermediate [I] and morning [M]) and MS pain were evaluated using multinomial logistic regression. To address the second aim, we included an interaction term (chronotype*mental distress, chronotype*insomnia) in the logistic model.

**Results:**

Compared to the M‐types, both the E‐ and I‐types had increased odds of suffering ‘disabling pain’ in the unadjusted model (odds ratio [OR] 1.79, 95% confidence interval [CI] 1.37–2.33; OR 1.54, 95% CI 1.29–1.84, respectively). However, the association remained statistically significant only after adjusting for all covariates amongst the I‐types (OR 1.39, 95% CI 1.15–1.67). Neither mental distress nor insomnia was found to modify the chronotype–MS pain association.

**Conclusions:**

The results highlight the importance of chronotypes for individuals’ MS health but suggest the presence of confounding factors in the interplay between these factors.

**Significance:**

This study shows that evening and intermediate chronotypes are associated with disabling MS pain, but that mental distress, insomnia and coexisting diseases also play a role in these associations.

## 1 INTRODUCTION

Musculoskeletal (MS) pain affects a substantial number of individuals worldwide (Cimmino et al., 2011; Jackson et al., 2015; Meucci et al., 2015). It causes considerable burden to working‐aged people and their employers by limiting work ability, increasing sickness absence rates and leading to early retirement (Bevan, 2015). It also reduces health‐related quality of life as a whole (Paananen et al., 2011). Despite voluminous research, we still lack effective treatment methods for MS pain (Foster et al., 2018). Preventive measures with adequate and accurate identification of risk factors are, therefore, crucial.

Sleep problems and mental distress have been recognized as one of the greatest risk factors for MS pain. They more than double the risk of chronic MS pain (Ortego et al., 2016; Skarpsno et al., 2021) and expose individuals with only occasional or acute MS pain to disabling chronic MS pain later in life (Holm et al., 2020; Young Casey et al., 2008). When co‐occurring with pain, sleep difficulties and mental distress contribute negatively to health‐related quality of life (Bair et al., 2008; Garnaes et al., 2021), health‐care use (Shmagel et al., 2016) and pain‐related disability (Bair et al., 2008; Holm et al., 2020; Saconi et al., 2021). It has been estimated that over 40% of individuals with chronic MS pain also suffer from sleep problems and/or mental distress (Artner et al., 2013; Bair et al., 2008; Mathias et al., 2018).

Both quality and quantity of sleep are domains that affect chronic MS pain (Generaal et al., 2017; Skarpsno et al., 2020, 2021). The aetiology of these sleep problems is likely to be multidimensional, with chronotype as one of the potential reasons for shortened and insufficient sleep (Kitamura et al., 2010; Merikanto et al., 2012). Chronotypes generally refer to variations in individuals’ timing of sleep‐wake rhythms. They are the behavioural manifestations of a 24‐h inner clock that regulates a myriad of physiological patterns, such as blood pressure and hormone levels (Horne & Östberg, 1976; Roenneberg & Merrow, 2016). It is likely that both genetics and environmental factors explain these variations (Barclay et al., 2014).

Characteristically, chronotypes can be placed into three different categories: morning (M), evening (E) or intermediate (I), depending on alertness levels during the day. Of these types, E‐type individuals (who are most alert in the evening) are most affected by sleep problems due to their inability to fully modify their inner clock rhythm to meet the requirements of society's social clock (Taillard et al., 2021). The disruptions in their sleep‐wake rhythms are one reason why they face more challenges than their counterparts with healthy lifestyles (Broms et al., 2012; Nauha et al., 2020) and good work performance (Räihä et al., 2021). In addition to sleep duration and quality, being an E‐type has also been associated with physical and mental illnesses, such as type 2 diabetes, arterial hypertension (Merikanto et al., 2013) and depression (Kitamura et al., 2010). In particular, E‐types report more mental disorder symptoms that require hospitalization (Merikanto & Partonen, 2021). As the 24‐h circadian rhythm seems to play a role in MS tissue physiology (Dudek & Meng, 2014; Morris et al., 2021), a rationale for further studies exists.

The potential relationships between chronotypes and MS pain remain poorly explored, and previous attention has mainly focused on fibromyalgia (Türkoğlu & Selvi, 2020) or MS pain with no assessment of pain frequency or pain‐related disability (Merikanto et al., 2014). Moreover, previous studies have been conducted in specific occupational populations (Zhang et al., 2018) or have neglected to evaluate the influence of sleep problems on the associations between MS pain and chronotypes (Merikanto et al., 2017).

To improve our understanding of the interplay between chronotypes and MS pain, the present study, which addressed a large population‐based birth cohort of working‐age Northern Finnish people, aimed to (1) examine the associations between chronotypes and MS pain, with special reference to disabling MS pain and (2) test whether mental distress and insomnia have a modifying role in these associations. Since E‐type people might be more sensitive to pain than M‐type people (Jankowski, 2013), we hypothesized that they have higher odds of suffering disabling MS pain than other chronotypes. Moreover, we hypothesized that mental distress and insomnia modify the associations between chronotype and MS pain.

## 2 METHODS

### 2.1 Study population

**FIGURE 1:**
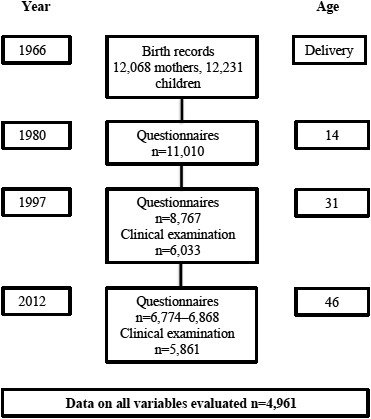
Flow chart of Northern Finland birth cohort 1966 data collection

The participants of the present study belonged to a large mother‐childbirth cohort, the Northern Finland Birth Cohort (NFBC1966). It originally consisted of pregnant women who lived in the Northern Finnish provinces of Oulu and Lapland in 1965–1966 and had an expected date of delivery between 1 January and 31 December 1966. A total of 12,068 mothers and their 12,231 children comprised the cohort base, covering 96% of births in the recruitment area. Since then, the children (members of the NFBC1966) have been approached regularly with questionnaires and clinical examinations (Figure 1; University of Oulu, 1966). In this study, we used cross‐sectional data collected from the 46‐year‐old cohort. They were sent several questionnaires on MS pain, demographics, potential covariates and chronotypes, and the response rate was 66%–67% (*n* = 6774–6868), depending on the questionnaire. Overall, 4961 of the participants had recorded data on all the variables evaluated in the final models. They gave their written permission for their data to be utilized for research, and the study was approved by the Ethics Committee of the Northern Finland Hospital District (94/2011).

### 2.2 MS pain

MS pain was assessed by asking: ‘Have you had any aches or pains in the following body parts in the last 12 months?’ (1) neck, (2) shoulder, (3) arms/elbows, (4) wrists/hands, (5) low back, (6) hips, (7) knees and (8) ankles/feet. The response options were (1) no, (2) on 1–7 days, (3) on 8–30 days, (4) on more than 30 days but not daily and (5) daily. All body parts of the MS systems were combined, and the overall frequency of pain was determined as the highest reported frequency category for any of the eight body parts. To study pain‐related disability over the preceding year, we asked the participants to rate their disability level on a scale of 0–10 (0 = no disability, 10 = extreme disability) in three subscales: (a) while working, (b) during leisure time and (c) while sleeping. The participants who answered six or over in any of the subscales were regarded as having a significant pain‐related disability, and those who answered under six in all subscales were regarded as not having a significant pain‐related disability (Boonstra et al., 2014). The pain frequency and disability data were combined to form three categories: (1) ‘no pain’ (= none of the aforementioned pains or some pain that lasted a maximum of 7 days), (2) ‘non‐disabling pain’ (= pain that lasted over 7 days but did not fulfil the criteria of disabling pain) and (3) ‘disabling pain’ (over 30 days of pain that caused significant disability).

### 2.3 Chronotypes

Information on chronotypes was collected via a short version of the Morningness‐Eveningness Questionnaire (sMEQ), translated in Finnish. It contained items 4, 7, 9, 15, 17 and 19 of the original MEQ (Horne & Östberg, 1976), which is known to correlate with the sleep‐wake cycles (Di Milia et al., 2013; Horne & Östberg, 1976; Taillard et al., 2003). It assesses, for example, hours of peak performance and ease of waking up in the morning. For the first four items, the response options were coded 1–4, for the fifth item 1–5 and for the last item 0, 2, 4 or 6, of which a total score variable, ranging from 5 to 27, was created in accordance with the MEQ scores. It has been estimated that the sum of these six items explains 83% of the variance in the total score of the original MEQ (Hätönen et al., 2008), and the internal consistency of sMEQ has been reported as good (Merikanto et al., 2012). The following cut‐offs for the Finnish population have been recommended: E‐type (5–12), I‐type (13–18) and M‐type (19–27) (Merikanto et al., 2012).

### 2.4 Potential confounding variables

#### 2.4.1 Insomnia

The five‐item Athens insomnia scale (AIS‐5) enquired about the participants’ sleep problems over the preceding month. AIS‐5 includes a four‐point Likert scale (0–3), with items on sleep induction, nocturnal awakenings, morning awakenings, total sleep duration and sleep quality. The participants were divided into two categories on the basis of the summed score (Enomoto et al., 2018; Soldatos et al., 2000): no insomnia (under four points) and insomnia (four or more points). AIS‐5 has good reliability and validity both in general (Soldatos et al., 2000) and in chronic pain populations (Enomoto et al., 2018).

#### 2.4.2 Sleep duration

To estimate sleeping hours, participants were asked to report their average hours and minutes of sleep time per night. The total sleeping time was dichotomized as (1) ‘recommended’ (7–9 h) and (2) ‘over or under recommended’, according to the National Sleep Foundation guidelines (Hirshkowitz et al., 2015).

#### 2.4.3 Smoking

The participants were categorized as non‐smokers, former smokers or current smokers on the basis of their responses to the questions ‘Have you ever smoked cigarettes?’ and ‘Do you currently smoke?’

#### 2.4.4 Mental distress

The widely used Hopkins Symptom Checklist‐25 (HSCL‐25) assesses symptoms of depression and anxiety. In the HSCL‐25, the participants rated themselves on each of 25 items on a scale of 1–4 (1 = not at all to 4 = extremely). According to the recommended cut‐off points (Veijola et al., 2003), the mean of the total score was dichotomized as 1.55 or over (‘severe mental distress’) versus under 1.55 (‘mild mental distress’). HSCL‐25 has shown to be a potential screening tool for psychiatric disorders (Veijola et al., 2003).

#### 2.4.5 Occupational status

Occupational status included 17 categories (Oura et al., 2020), from which we created three separate categories: (1) employed, (2) unemployed or retired and (3) others (including students, participants on parental or sabbatical leave, homemakers or those doing something other than any of the aforementioned).

#### 2.4.6 Education level

Education level was classified as (1) compulsory or no basic education, (2) secondary (upper secondary or vocational school) and (3) tertiary (university or university of applied sciences) on the basis of the participants’ responses to the questions ‘What is your basic education?’ and ‘What is your vocational education?’

#### 2.4.7 Number of coexisting diseases

The NFBC1966 members were asked to report the potential existence of 75 symptoms, diseases or traumas diagnosed by a medical doctor. They were also asked about their weight and height, from which body mass index (BMI) was calculated (kg/m^2^). In addition to obesity (BMI > 30 kg/m^2^), the following chronic diseases were statistically significantly associated with both MS pain and chronotypes in this dataset after controlling for the remaining diseases and were, therefore, included in the sum variable: hypertension, diabetes, hypo‐ and hyperthyroidism, sleep apnoea, skin diseases (skin cancer excluded), migraine and psychoactive substance use disorder. As such, the number of coexisting diseases had a theoretical range of 0–8.

### 2.5 Statistical analysis

Statistical analysis was performed using SPSS version 27.0. We set the threshold for statistical significance at *p* = 0.05. Cross‐tabulations with the chi‐square test were utilized to estimate the differences between the variable distributions of the MS pain categories. Multinomial logistic regression analysis with odds ratios (ORs) and 95% confidence intervals (CIs) was conducted to evaluate (1) the univariate associations of the confounding variables with the exposure (chronotype) and the outcome (MS pain category), to rule out unnecessary covariate candidates that were not associated with the exposure or the outcome and (2) the associations between the exposure and the outcome, before and after adjustments. As for chronotypes, M‐type was used as the reference category. The selection of eight chronic diseases included in the ‘number of coexisting’ variable was also based on a statistically significant association with both MS pain and chronotypes in this dataset, after controlling for other diseases. All the confounding variables included were associated with both chronotype and MS pain (Supplements 1 and 2). To estimate the potential confounding effects of different confounding variable patterns on the chronotype–MS pain associations, we built four models: (1) unadjusted, (2) adjusted for sociodemographics (sex, occupational status and education level), (3) additionally adjusted for lifestyle factors (smoking and sleep duration) and (4) additionally adjusted for comorbidities (insomnia, mental distress and number of coexisting diseases). To further assess the potential modifying role of mental distress and insomnia in the chronotype–MS pain association, we included an interaction term (chronotype*mental distress, chronotype*insomnia) in the corresponding logistic model. We evaluated potential multicollinearity using variance inflation factor (VIF) values and noticed no significant collinearity between the explanatory factors, including chronotype and confounding variables (all VIFs < 4; Miles & Shevlin, 2000).

## 3 RESULTS

Overall, of the 4961 NFBC1966 members with MS pain data available, 27% had ‘disabling pain’ and 54% reported ‘non‐disabling pain’, whereas 19% were placed in the ‘no pain’ category (Table 1). A higher proportion of participants in the ‘disabling pain’ category than in the other pain categories were women, non‐employed, had compulsory or no basic education, slept under/over recommended, had insomnia and severe mental distress, smoked and had a higher number of coexisting diseases (*p* < 0.001). Half of the participants (51%) without MS pain were M‐types, and fewer of them were in other pain categories (42% in ‘non‐disabling pain’ and 40% in ‘disabling pain’ categories, *p* < 0.001). The percentage of E‐types was the highest in the ‘disabling pain’ category (15% vs 11% and 12% in the ‘no pain’ and ‘non‐disabling pain’ categories, respectively; *p* < 0.001).

**TABLE 1:** Demographics of the Northern Finland birth cohort 1966 at 46 years, stratified by musculoskeletal pain status

	Musculoskeletal pain status				
	‘No pain’ (*n* = 959) % (*n*)	‘Non‐disabling pain’ (*n* = 2677) % (*n*)	‘Disabling pain’ (*n* = 1325) % (*n*)	Total (*n* = 4961) % (*n*)	*p* value (x^2^)
Sex					<0.001
Men	56.5 (542)	45.4 (1215)	37.8 (501)	45.5 (2258)	
Women	43.5 (417)	54.6 (1462)	62.2 (824)	54.5 (2703)	
Chronotype					<0.001
Evening	10.9 (105)	11.8 (315)	15.2 (201)	12.5 (621)	
Intermediate	37.6 (361)	45.9 (1230)	45.0 (596)	44.1 (2187)	
Morning	51.4 (493)	42.3 (1132)	39.8 (528)	43.4 (2,153)	
Insomnia					<0.001
No	77.4 (742)	68.6 (1837)	50.6 (671)	65.5 (3250)	
Yes	22.6 (217)	31.4 (840)	49.4 (654)	34.5 (1711)	
Sleep duration					<0.001
Recommended	82.1 (787)	81.2 (2175)	75.7 (1003)	79.9 (3965)	
Under or over recommended	17.9 (172)	18.8 (502)	24.3 (322)	20.1 (996)	
Smoking					<0.001
Non‐smokers	50.0 (480)	47.6 (1275)	40.8 (540)	46.2 (2295)	
Former smokers	26.0 (249)	26.8 (717)	29.6 (392)	27.4 (1358)	
Current smokers	24.0 (230)	25.6 (685)	29.6 (393)	26.4 (1308)	
Mental distress					<0.001
Mild	90.4 (867)	81.7 (2188)	69.7 (924)	80.2 (3979)	
Severe	9.6 (92)	18.3 (489)	30.3 (401)	19.8 (982)	
Occupational status					<0.001
Employed	84.4 (809)	84.8 (2271)	78.0 (1033)	82.9 (4113)	
Unemployed or retired	7.5 (72)	5.9 (159)	9.1 (121)	7.1 (352)	
Other	8.1 (78)	9.3 (247)	12.9 (171)	10.0 (496)	
Education level					<0.001
Compulsory or no basic education	7.0 (67)	6.4 (172)	9.3 (123)	7.3 (362)	
Secondary	60.9 (584)	65.7 (1759)	67.0 (888)	65.1 (3231)	
Tertiary	32.1 (308)	27.9 (746)	23.7 (314)	27.6 (1368)	
Number of coexisting diseases					<0.001
0	49.5 (475)	41.8 (1118)	33.8 (448)	41.1 (2041)	
1	32.8 (315)	34.2 (917)	33.4 (443)	33.8 (1675)	
2	12.3 (118)	16.3 (437)	22.1 (293)	17.1 (848)	
3	3.9 (37)	5.9 (157)	7.1 (94)	5.8 (288)	
4	1.3 (12)	1.5 (40)	2.6 (34)	1.7 (86)	
5	0.2 (2)	0.3 (7)	0.7 (9)	0.4 (18)	
6		0 (0)	0.2 (3)	0.1 (3)	
7		0 (1)	0.1 (1)	0 (2)	

Table 2 characterizes the associations between the chronotypes and MS pain. In the unadjusted model, both E‐ and I‐types were found to relate to both MS pain categories. The highest ORs were observed between the E‐type and the ‘disabling pain’ category (OR 1.79, 95% CI 1.37–2.33). The associations between the I‐type and the ‘disabling pain’ and ‘non‐disabling pain’ categories remained statistically significant after adjusting for sociodemographics (sex, occupational status and education level), lifestyle factors (sleep duration and smoking) and comorbidities (insomnia, mental distress and number of coexisting diseases) (OR 1.39, 95% CI 1.15–1.67; OR 1.43, 95% CI 1.22–1.68, respectively). The associations between the E‐type and the ‘disabling pain’ and ‘non‐disabling pain’ categories were also statistically significant in the models adjusted for sociodemographics and lifestyle factors, but they attenuated to non‐significant after further adjustment for comorbidities (OR 1.15, 95% CI 0.86–1.52; OR 1.10, 95% CI 0.85–1.42, respectively). The interaction terms (chronotype*mental distress and chronotype*insomnia) were not statistically significant in any model (*p* > 0.05; data not shown).

**TABLE 2:** Associations of chronotype with musculoskeletal pain status (*n* = 4961)

	Musculoskeletal pain status		
	‘Disabling pain’	‘Non‐disabling pain’	‘No pain’
Chronotypes			
Unadjusted			
Evening	**1.79** (1.37–2.33) (*n* = 201)	**1.31** (1.02–1.70) (*n* = 315)	1 (*n* = 105)
Intermediate	**1.54** (1.29–1.84) (*n* = 596)	**1.48** (1.27–1.74) (*n* = 1230)	1 (*n* = 361)
Morning	1 (*n* = 528)	1 (*n* = 1132)	1 (*n* = 493)
Adjusted for sociodemographics^a^			
Evening	**1.75** (1.34–2.29) (*n* = 201)	**1.31** (1.03–1.68) (*n* = 315)	1 (*n* = 105)
Intermediate	**1.59** (1.33–1.91) (*n* = 596)	**1.51** (1.29–1.78) (*n* = 1230)	1 (*n* = 361)
Morning	1 (*n* = 528)	1 (*n* = 1132)	1 (*n* = 493)
Adjusted for sociodemographics and lifestyle factors^b^			
Evening	**1.65** (1.26–2.17) (*n* = 201)	**1.29** (1.01–1.66) (*n* = 315)	1 (*n* = 105)
Intermediate	**1.56** (1.30–1.88) (*n* = 596)	**1.51** (1.29–1.77) (*n* = 1230)	1 (*n* = 361)
Morning	1 (*n* = 528)	1 (*n* = 1132)	1 (*n* = 493)
Adjusted for sociodemographics, lifestyle factors and comorbidities^c^			
Evening	1.15 (0.86–1.52) (*n* = 201)	1.10 (0.85–1.42) (*n* = 315)	1 (*n* = 105)
Intermediate	**1.39** (1.15–1.67) (*n* = 596)	**1.43** (1.22–1.68) (*n* = 1230)	1 (*n* = 361)
Morning	1 (*n* = 528)	1 (*n* = 1132)	1 (*n* = 493)

Note

Odds ratios with 95% confidence intervals. Statistically significant values are bolded.

^a^ Sociodemographics = sex, occupational status and education level.

^b^ Lifestyle factors = sleep duration and smoking.

^c^ Comorbidities = insomnia, mental distress and number of coexisting diseases.

## 4 DISCUSSION

This large cohort study addressed the potential associations between chronotypes and disabling MS pain amongst the general working‐age population. The study found that the E‐ and I‐types, in contrast to the M‐types, had higher odds of suffering ‘disabling pain’. However, after adjustments for sex, sleep duration, smoking, occupational status, education level, number of diseases, insomnia and mental distress, only the relationship between the I‐type and ‘disabling pain’ remained statistically significant. Neither mental distress nor insomnia was found to modify the chronotype–MS pain association.

One‐fifth of the 46‐year‐old working‐age population had no pain, while more than one‐quarter reported MS pain that had lasted over a month over the preceding year and had caused notable disability. Although the definition of the ‘disabling pain’ group does not fully equate with the definition of chronic pain (pain lasting longer than 3 months; Treede et al., 2015), 50% of the participants in this group struggled with daily pain (data not shown). A review by Meucci et al. (2015) estimated that the worldwide prevalence of chronic low back pain was approximately 20% amongst adults aged between 20 and 59. In turn, a slightly older review by Cimmino et al. (2011) found that between 11% and 24% of individuals had chronic and multisite MS pain. With respect to the frequency of chronic disabling pain, estimates have generally ranged between 11% and 14% in different studies (Arnow et al., 2006; Fayaz et al., 2016). Our results are in line with previous assessments.

In the present study, the I‐types were more likely than the M‐types to suffer from ‘disabling pain’. A Finnish study by Merikanto et al. (2014) found I‐types to have higher odds of back pain, but not spinal disease diagnosed or treated by a doctor. No associations between the I‐type and hospital treatment due to articular or spinal diseases were recorded in further analyses (Merikanto et al., 2017). These observations are somewhat contradictory to the present ones, although partly parallel, as we also observed the I‐type to be related to ‘non‐disabling pain’. The I‐type category is likely to include participants who are afternoon types and who have both M‐ and E‐type traits but do not completely fulfil their definitions (Horne & Östberg, 1976). Perhaps, they experience more disruptions to circadian rhythm, e.g., due to shift work, than the M‐types (Räihä et al., 2021), which expose MS tissues to degenerative changes (Gossan et al., 2015; Morris et al., 2021). Unquestionably, the underlying mechanisms for I‐types being the ‘high’ risk group for disabling pain remain to be explored.

Based on the current data, eveningness was associated with ‘disabling pain’, although the association was statistically non‐significant after adjustments. In a gradually adjusted model, we noticed that adding insomnia, mental distress and the number of coexisting diseases to the model diluted the significant association. In their studies, Merikanto et al. (2014, 2017) found that the E‐type was significantly related to reporting back pain and seeking treatment for it. Similarly, Zhang et al. (2018) suggested an association between the E‐type and work‐related MS pain. Merikanto et al.’s later study (2017) noticed that eveningness protected patients from hospitalization due to arthropathies. The reports are in line with ours in some ways, but contradictory in others, as the E‐type–MS pain association remained significant after adjustments in these studies. However, none of the publications adjusted the analyses for coexisting diseases or both sleep problems and mental health problems. Merikanto et al. (2014) took depressive symptoms and insufficient sleep into account but did not add both to the same model. The current study adds to the evidence highlighting the presence of confounding factors in the E‐type–MS pain association.

E‐types have been previously acknowledged to be prone to depression, insomnia and chronic diseases (Kivelä et al., 2018; Merikanto et al., 2013), whereas a large study by Stickley et al. (2019) claims that individuals with sleep problems and depression are more likely to suffer pain than their counterparts with only sleep problems, providing some support for the current observations. However, the explanatory pathways of the chronotype–MS pain association warrant further study, particularly of the interplay between insomnia, mental health problems and disease burden. Nevertheless, it seems that neither mental distress nor insomnia has a modifying role in the association between chronotype and MS pain.

Overall, as both the I‐ and E‐types had systematically higher odds of ‘disabling pain’ and ‘non‐disabling pain’ than the M‐types, our findings are indicative of the seemingly protective role of the M‐types in pain. M‐types appear to have healthier lifestyles and fewer mental health problems (Merikanto et al., 2014; Räihä et al., 2021), which, along with the potentially lowered discordance between their inner clock and society's social clock, are likely to explain the favourable existence of the M‐chronotype in MS health (Foster et al., 2013).

The main strength of the current study is that it is based on a large sample of working‐age individuals who had pain‐related frequency and disability data available. Importantly, the analysis was based on a general population sample, which increased the generalizability of our findings.

The following limitations should still be noted when interpreting our results. First, the data relied on self‐report data, which is acknowledged to be vulnerable to recall bias, and more objective measurements might have influenced the estimates, on, for example, chronotype (24‐h monitoring of body temperature has been suggested; Yamakoshi et al., 2013). However, with our large sample size, methods other than survey‐based data collection would have been challenging and expensive to conduct. Moreover, objective measures for much of the information addressed, such as pain, are lacking. Second, because of the cross‐sectional setting, causal relationships cannot be determined. To clarify the directions in chronotype–MS pain relationships, studies using a longitudinal approach are called for. Third, as this was an observational study, we might not have captured some uncontrolled variables that affected the observed associations.

## 5 CONCLUSIONS

Using general population data, this study demonstrated that both eveningness and intermediate chronotypes amongst working‐age individuals were related to ‘disabling pain’. However, the association between eveningness and ‘disabling pain’ attenuated after adjustment for insomnia, mental distress and a number of coexisting diseases. The results highlight the importance of chronotypes in individuals’ MS health. E‐types and I‐types should receive special attention for MS pain. However, other factors, especially those related to health, are likely to have a role in the interplay between chronotypes and MS pain, and thus, further research is warranted.

## CONFLICTS OF INTEREST

None to disclose.

## AUTHOR CONTRIBUTION

EH, PO and MP designed the study and interpreted the results. EH drafted the first manuscript and performed the statistical analyses. PO, MP, TK and JK participated in the writing process and critically revised the manuscript. All the authors have discussed the results, commented on the manuscript and approved its final version.

## Supporting information

Table S1

Table S2
