# Supplementary material for: Chronotypes and disabling musculoskeletal pain: A Finnish birth cohort study
Source: Eur J Pain. 2022 Mar 18;26(5):1069–78. doi: 10.1002/ejp.1931 (PMC9310771; doi:10.1002/ejp.1931)
Supplement: Supplementary file 1 — Table S1 [file EJP-26-1069-s002.docx]

| Supplement 1. Univariate associations of covariate candidates with musculoskeletal pain status. Odds ratios with 95% confidence intervals. | | | |
| --- | --- | --- | --- |
|  | **Musculoskeletal pain status** | | |
|  | ’Disabling pain’ | ’Non-disabling pain’ | ’No pain’ |
| *Sex* |  |  |  |
| Women | 2.14 (1.81–2.53) | 1.56 (1.35–1.82) | 1 |
| Men | 1 | 1 |  |
| *Insomnia* |  |  |  |
| Yes | 3.33 (2.77–4.01) | 1.56 (1.32–1.86) | 1 |
| No | 1 | 1 |  |
| *Sleep duration* |  |  |  |
| Under or over recommended | 1.47 (1.19–1.81) | 1.06 (0.87–1.28) | 1 |
| Recommended | 1 | 1 |  |
| *Smoking* |  |  |  |
| Current smoker | 1.52 (1.24–1.86) | 1.12 (0.93–1.35) | 1 |
| Former smoker | 1.40 (1.15–1.71) | 1.08 (0.91–1.30) | 1 |
| Non-smoker | 1 | 1 |  |
| *Mental distress* |  |  |  |
| Severe | 4.09 (3.20–5.22) | 2.11 (1.66–2.67) | 1 |
| Mild | 1 | 1 |  |
| *Occupational status* |  |  |  |
| Other | 1.72 (1.29–2.28) | 1.13 (0.86–1.47) | 1 |
| Unemployed or retired | 1.32 (0.97–1.79) | 0.79 (0.59–1.05) | 1 |
| Employed | 1 | 1 |  |
| *Education level* |  |  |  |
| Compulsory or no basic education | 1.80 (1.29–2.52) | 1.06 (0.78–1.45) | 1 |
| Secondary | 1.49 (1.24–1.80) | 1.24 (1.06–1.46) | 1 |
| Tertiary | 1 | 1 |  |
| *Number of co-existing diseases* | 1.47 (1.35–1.60) | 1.21 (1.11–1.31) | 1 |
