# Supplementary material for: Chronotypes and disabling musculoskeletal pain: A Finnish birth cohort study
Source: Eur J Pain. 2022 Mar 18;26(5):1069–78. doi: 10.1002/ejp.1931 (PMC9310771; doi:10.1002/ejp.1931)
Supplement: Supplementary file 2 — Table S2 [file EJP-26-1069-s001.docx]

| Supplement 2. Univariate associations of covariate candidates with chronotypes. Odds ratios with 95% confidence intervals. | | | |
| --- | --- | --- | --- |
|  | **Chronotypes** | | |
|  | Evening | Intermediate | Morning |
| *Sex* |  |  |  |
| Women | 1.29 (1.08–1.55) | 1.00 (0.89–1.13) | 1 |
| Men | 1 | 1 |  |
| *Insomnia* |  |  |  |
| Yes | 2.16 (1.80–2.60) | 1.33 (1.17–1.51) | 1 |
| No | 1 | 1 |  |
| *Sleep duration* |  |  |  |
| Under or over recommended | 1.57 (1.27–1.93) | 1.11 (0.96–1.29) | 1 |
| Recommended | 1 | 1 |  |
| *Smoking* |  |  |  |
| Current smoker | 1.51 (1.22–1.87) | 1.13 (0.97–1.30) | 1 |
| Former smoker | 1.60 (0.93–1.45) | 1.12 (0.97–1.29) | 1 |
| Non-smoker | 1 | 1 |  |
| *Mental distress* |  |  |  |
| Severe | 3.04 (2.47–3.73) | 1.62 (1.38–1.90) | 1 |
| Mild | 1 | 1 |  |
| *Occupational status* |  |  |  |
| Other | 1.46 (1.10–1.94) | 1.14 (0.93–1.39) | 1 |
| Unemployed or retired | 1.48 (1.08–2.04) | 1.01 (0.80–1.28) | 1 |
| Employed | 1 | 1 |  |
| *Education level* |  |  |  |
| Compulsory or no basic education | 0.94 (0.67–1.32) | 0.70 (0.54–0.90) | 1 |
| Secondary | 0.69 (0.57–0.85) | 0.80 (0.70–0.92) | 1 |
| Tertiary | 1 | 1 |  |
| *Number of co-existing diseases* | 1.42 (1.31–1.54) | 1.11 (1.05–1.18) | 1 |
